# On Modeling HIV and T Cells In Vivo: Assessing Causal Estimators in Vaccine Trials

**DOI:** 10.1371/journal.pcbi.0020064

**Published:** 2006-06-16

**Authors:** W. David Wick, Peter B Gilbert, Steven G Self

**Affiliations:** Statistical Center for HIV and AIDS Research and Prevention, Fred Hutchinson Cancer Research Center, Seattle, Washington, United States of America; ETH, Zürich, Switzerland

## Abstract

The first efficacy trials—named STEP—of a T cell vaccine against HIV/AIDS began in 2004. The unprecedented structure of these trials raised new modeling and statistical challenges. Is it plausible that memory T cells, as opposed to antibodies, can actually prevent infection? If they fail at prevention, to what extent can they ameliorate disease? And how do we estimate efficacy in a vaccine trial with two primary endpoints, one traditional, one entirely novel (viral load after infection), and where the latter may be influenced by selection bias due to the former? In preparation for the STEP trials, biostatisticians developed novel techniques for estimating a causal effect of a vaccine on viral load, while accounting for post-randomization selection bias. But these techniques have not been tested in biologically plausible scenarios. We introduce new stochastic models of T cell and HIV kinetics, making use of new estimates of the rate that cytotoxic T lymphocytes—CTLs; the so-called killer T cells—can kill HIV-infected cells. Based on these models, we make the surprising discovery that it is not entirely implausible that HIV-specific CTLs might prevent infection—as the designers explicitly acknowledged when they chose the endpoints of the STEP trials. By simulating thousands of trials, we demonstrate that the new statistical methods can correctly identify an efficacious vaccine, while protecting against a false conclusion that the vaccine exacerbates disease. In addition to uncovering a surprising immunological scenario, our results illustrate the utility of mechanistic modeling in biostatistics.

## Synopsis

In traditional biostatistics, mechanistic modeling of the relevant biology usually plays no role, because regulatory agencies will not, quite understandably, license vaccines or drugs on the basis of theories. But the second wave of trials of HIV vaccines will test two conjectures simultaneously. The theoretical possibility that these new, nonclassical, T cell–directed vaccines will prevent some infections while only ameliorating disease in others required biostatisticians to invent new ways of estimating vaccine efficacy. When only the one traditional endpoint—infection—is analyzed, the randomization to vaccine or placebo groups protects against bias. But the new techniques required input from experts on the plausible range of bias introduced by post-randomization selection (by infected state) for the second analysis. Here mechanistic modeling can play a role in evaluating the statistical methodology in biologically plausible settings. By simulating thousands of trials using their models, Wick, Gilbert, and Self were able to demonstrate that the methods protected from falsely concluding a harmful effect of the vaccine on disease. They also noted that the so-called killer T cells, as opposed to antibodies raised by a traditional vaccine, may actually be able to prevent some infections—a conclusion rather surprising for most immunologists and virologists, but which had to be allowed for when designing the vaccine trials.

## Introduction

The first generation of vaccines against the human immunodeficiency virus (HIV), designed to prevent HIV acquisition by stimulating neutralizing antibodies, failed to protect in efficacy trials [1]. Second-generation vaccines have been designed to elicit HIV-specific cellular immune responses [2]. These candidates are supported by evidence that so-called killer T cells—the cytotoxic T lymphocytes (CTLs), bearing the CD8 membrane-molecule, that can react to and kill infected target (IT) cells—play a crucial role in controlling HIV infection [3–11].

The first efficacy trial, named STEP, of a T cell–directed HIV vaccine began in December 2004; it is being conducted by Merck Research Laboratories in collaboration with the HIV Vaccine Trials Network and the Division of AIDS at the US National Institutes of Health. The candidate vaccine (MRKAd5) consists of three vectors that can ferry HIV proteins into human cells (adenovirus serotype-5, encoding the HIV *gag, pol,* and *nef* genes, respectively). The vaccine elicits broad T cell responses in a majority of vaccinated HIV-uninfected adults [12]. The STEP trial will randomize 3,000 HIV uninfected volunteers to receive MRKAd5 or placebo in a 1:1 ratio and follow participants until 100 HIV infections occur. Mehrotra, Li, and Gilbert [13] provide details of this trial. A second STEP trial of MRKAd5 with a nearly identical design will begin in South Africa in 2006.

The co-primary endpoints of the STEP trials are HIV infection and a clinical measure of disease: setpoint viral load. The terminology reflects the typical course of HIV disease, which appears first as a flu-like illness (called primary viremia and lasting for about a month), progresses through a stable, asymptomatic phase (which can last ten years or more), then (if untreated) progresses to AIDS. The viral load is typically measured in blood drawn sometime after the primary stage (and expressed as virions per milliliter, for example). Even without preventing infection, a vaccine that suppresses viral load could confer a benefit to the individual, by slowing progression to AIDS [14,15] and preventing the need for antiretroviral treatment; and to the community by reducing HIV transmission [16,17]. The second primary analysis of STEP compares viral load setpoints among HIV-infected subjects in the vaccine and placebo groups.

Besides the unprecedented nature of the trials (the first to test a T cell vaccine in humans, to our knowledge), the nontraditional design presented a statistical challenge. Because the subjects included in the viral-load comparison are determined by a post-randomization event, HIV infection, the analysis is susceptible to selection bias [18,19]. Specifically, a conventional analysis of viral load differences would not uniquely assess a causal effect of the vaccine, but rather a mixture of causal vaccine-effect and the effects of variables correlated with viral load (e.g., host genetics). The latter may be unevenly distributed between the infected-vaccinated and infected-placebo groups. For example, selection bias would occur if the vaccine protects from HIV infection only vaccinees with a relatively strong immune system, which implies the infected vaccine group would be weaker immunologically than the infected placebo group. Consequently, even if the vaccine has no causal effect on viremia, the viral loads in the vaccine group would be expected to be higher than those in the placebo group. Failing to account for this selection bias would lead to the incorrect inference that the vaccine harmfully increases viral load.

In preparation for the analysis of the STEP trials, Gilbert, Bosch, and Hudgens (GBH) [20] and other investigators [21,22] developed statistical techniques for assessing a vaccine effect on viral load that allow for plausible (expert-specified) levels of selection bias. However, these papers did not consider underlying biological mechanisms that could account for causal vaccine effects. Rather, they simulated arbitrary effects and studied the purely statistical operating characteristics of the methodology.

To evaluate the statistical methods in a more biologically relevant manner, we consider here various mechanistic hypotheses for vaccine effects. At their present stage of development, mathematical models of HIV infection and the immune system have made few compelling predictions, primarily because of uncertainty about which are the most important mechanisms and the values of rate-constants. Nevertheless, models have attained enough maturity that they can quantitatively reproduce the drop in primary viremia after appearance of HIV-specific CTLs, the lag between peak viremia and peak immune response, the formation of a steady state, and other aspects of HIV infection [23–26]. In addition, we can exploit a recent estimate of the rate at which HIV-specific CTLs can kill HIV-infected cells [11]. Building on these developments, we constructed new stochastic models of primary infection and the immune response and made a surprising observation: it does not appear implausible (at least in models) that CTLs might abort an HIV infection. By using infection as a co-primary endpoint with setpoint viral load, the designers of the STEP trials explicitly acknowledged this possibility.

For purposes of discussion, let us distinguish prevention from eradication of infection. By *prevention* we will mean either that no productively infected target (PIT) cells arise at all, or the pool does not expand beyond some small number of cells; afterward, it is driven to extinction. The number of PITs at any time is insufficient to cause disease. (For HIV, PITs are primarily two other kinds of immune-system cells: T cells carrying the CD4 surface molecule, and macrophages. We include the modifier *productive* in defining PIT because infected T cells may revert to an unproductive, or latent, state.) Such a favorable outcome might be described as a transient infection or as sterilizing immunity, depending on which assays are employed to detect exposure. (Finding HIV-derived DNA in latently infected CD4 T cells, or HIV-specific CD8+ memory cells, would point to the former.) *Eradication* we restrict, consistent with common usage, to clearing the infection after primary, symptomatic viremia.

Because CD8 T cells require a priming (activation) step and an expansion (proliferation) period before they can clear infected cells, most immunologists regard preventing infection—as we have defined it—to be unlikely [27]. That vaccines against simian immunodeficiency virus (SIV) have not prevented infection may be a consequence of the large inoculums used in the experiments [23]. More relevant to HIV is the observation of T cell memory to HIV antigens in frequently exposed but seronegative sex workers in Kenya [28]. One mechanistic interpretation of this finding is that a productive cellular infection was initially established, but was either cleared by CTLs or went extinct due to chance (consistent with our models; see below). The transient infection left behind a small pool of latently infected, resting CD4 T cells that, due to occasional activation events, continuously expose the immune system to HIV antigens, maintaining CTL memory. However, the investigators could not prove that the HIV-specific CTLs actually protected these women. But vaccine-derived or adoptively transferred CTLs have prevented infection by other viruses; in particular, Sendai [29] and Ebola [30] in mice. However we may judge the plausibility of prevention by T cells, as we are interested here in the impact on statistical estimation, we have to incorporate some biological mechanism for it into our simulations.

We have combined stochastic and deterministic models so as to simulate the impact of T cells both on the probability of infection given exposure and on viral load assuming infection, in vaccinated or unvaccinated subjects. Of course, such a concatenation requires more hypotheses, in particular about vaccine action. Again, because of the extent of uncertainty about these mechanisms, we do not claim to predict the outcome of vaccine trials. Rather, the models provide cases where an influence of selection bias is present or absent, and when present of a magnitude resulting from biological scenarios rather than ad hoc assumptions. We can then generate thousands of hypothetical STEP trials, and put the GBH method to the test.

### Statistical Assessment of Causal Vaccine Efficacy of a T Cell Vaccine

In the STEP trials, two vaccine efficacy parameters will be assessed; one-minus-relative-risk of HIV infection, and the difference (placebo–vaccine) in mean viral load setpoint of HIV-infected subjects, where setpoint viral load is defined as the average of two log10 plasma HIV RNA levels measured at month 2 and 3 visits after diagnosis of HIV infection. The data will be analyzed using an adaption of the GBH method, which estimates the causal vaccine effect on viral load while accounting for plausible levels of selection bias. This technique was developed from the potential outcomes framework for causal inference [31,32]. In this framework, each trial participant has two potential HIV infection outcomes: one under assignment to vaccine and one under assignment to placebo. Following GBH, a causal effect on viral load can be defined for the subpopulation of always-infected subjects who would become HIV-infected regardless of randomization to vaccine or placebo. Such an effect is causal because the always-infected subpopulations of the two study arms have identical characteristics except for the vaccine/placebo assignment, and therefore observed differences are directly attributable to vaccination. The methods estimate the average causal effect (*ACE*) of vaccine on viral load, equal to the difference in mean setpoint viral loads (placebo–vaccine) for the always-infected subpopulation. The fundamental difficulty in evaluating the *ACE* is the lack of knowledge about which infected subjects are in the always-infected group—knowing this would require data on subjects' HIV infection outcome both under assignment to vaccine and under assignment to placebo, but for each subject only one of these outcomes is observed.

To address the identifiability problem, GBH made the simplifying assumption that the vaccine does not increase the risk of infection for any subject, and posited a model for whether an infected placebo recipient with setpoint viral load *Y* would have been infected had they been assigned vaccine. This model is indexed by a sensitivity parameter *β,* which is the log odds ratio of infection under assignment to vaccine comparing two infected placebo recipients with setpoint viral loads *Y* and *Y* − 1. A value *β* = 0 reflects the case of no selection bias, in which case the naive analysis assesses a causal vaccine effect, and positive (negative) values of *β* reflect selection bias such that the odds of infection is higher (lower) for larger setpoint viral loads *Y.* The parameter *β* is fixed by the investigator at each possible value within a plausible range, and for each *β,* GBH provided procedures for estimating *ACE*(*β*) with a confidence interval. See Materials and Methods for the mathematical details. For the first STEP trial, a panel of ten experts proposed a plausible range for *β* of log(.5) to log(7); see Discussion section. To be cautious, our analysis will estimate *ACE*(*β*) over a somewhat wider range for *β,* namely log(0.37) to log(7.4); i.e., *β* ∈ [−1,2].

### Mechanistic Modeling of HIV, CTLS, and Vaccine Effects

Mathematical models of HIV can be divided into two classes: the whole-body, deterministic, and the small-volume, stochastic. The former models are appropriate in a discussion of steady-state viral load, but inappropriate for treating the early events in infection [23]. The latter class is capable of addressing random outcomes after exposure.

#### Predicting viral load: The whole-body, deterministic model.

For this model class, both the infection process and the immune system are assumed to have large numbers of cells in each compartment, meaning a subset of cells sharing all transition rates. The usage is, however, not bio-geographic (as in tissue compartments); rather, cells are assumed to mix uniformly in the body. The dynamics are mass-action and determined by parameters that express the rate, per unit time per cell, of a particular transition. In Materials and Methods, we display all possible transitions and label the corresponding rate-constants (see Table 1); the rates themselves are given in Table 2. The ODEs (ordinary differential equations) used for simulating are consequences of the rates (see Discussion). Here we describe the processes informally.

**Table 1:**
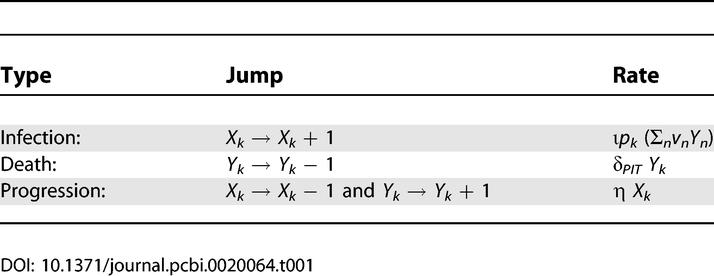
Transitions of the Infection Process

**Table 2:**
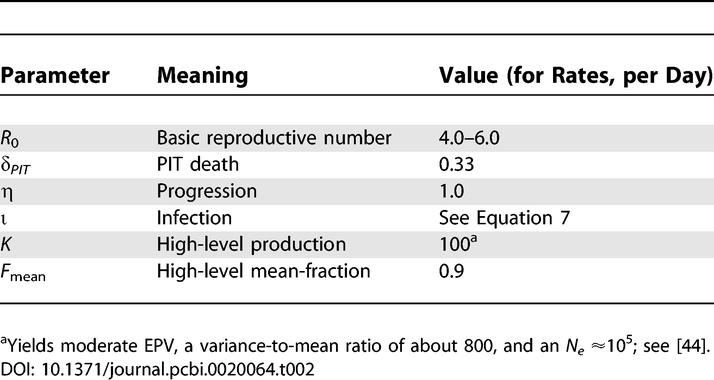
Rates in the Infection Process

For the infection side, a target cell passes through two stages, the eclipse or IT phase, which lasts about a day, and the PIT phase, whose length depends on the intensity of killing by CTLs. Virions are not represented explicitly in the model, because of their short lifetime in vivo (hours at most); nor are uninfected target cells, a consequence of the belief that the cellular immune response—as opposed to target-cell limitation—controls the infection [9]. Hence, the birth rate of a new IT is proportional to the number of PITs existing at that time. The proportionality rate-constant is determined by the basic reproductive number, *R*_0_ (see Equation 7).

On the immune system side, the kinetic model for T cell dynamics, introduced in [24], implements the programmed-proliferation scenario, in which CD8+ T cells, once activated, proceed through a sequence of doublings (at least eight) before reverting to resting status or to being eliminated by apoptosis. The model contains both naïve and memory cells. Memory cells are activated faster, pass more quickly through the cell cycle, and acquire effector status faster than naïve cells. We may modify the basic model to incorporate an HIV-induced “memory defect” or “killing defect,” (see [24]). (This option reflects the opinion that HIV infection damages the immune system, particularly by killing CD4 T cells that assist CTLs and other immune system cells in maturing and functioning.) Many other aspects of CTLs, such as epitope specificity, affect their potency in suppressing HIV [33], but are not included in our simplified model. See Modeling the Cellular Immune Response in Materials and Methods and Table 3 for the process and Table 4 for the rates.

**Table 3:**
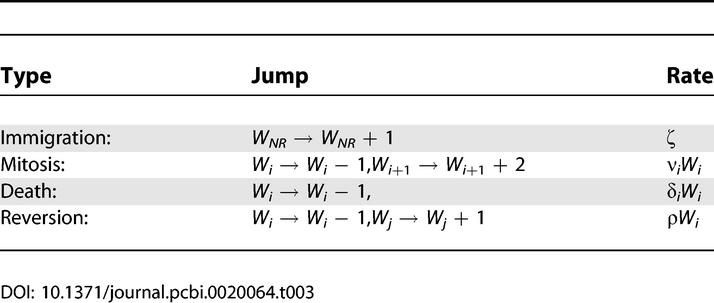
Transitions of the HIV-Specific, CD8 Cellular-Immune-System Model

**Table 4:**
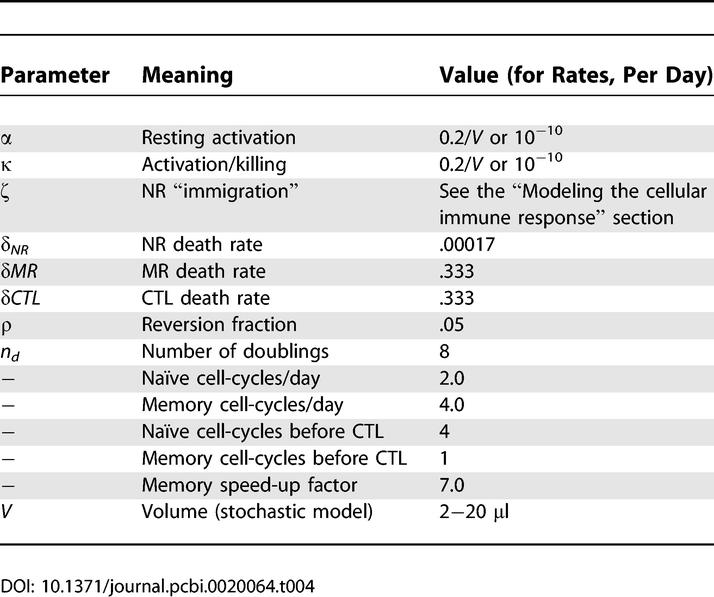
Rates in the HIV-Specific, CD8 Cellular-Immune-System Model

Figure 1 shows a choice of parameters yielding peak viremia in a little more time than a month, and steady state in three months. On a longer timescale, the model displays a stable steady state (whose existence can be verified mathematically). To create Figure 1, the resting-cell activation and CTL activation-and-killing parameters (denoted *α* and *κ* in Materials and Methods) were set to about 10^−10^, obtained by scaling the value estimated in [11]. This work analyzed data from in vitro studies of CTLs suppressing HIV replication in cell lines, as well as adoptive-transfer studies of CTLs in HIV-infected patients, and estimated a combined activation and killing parameter at about 0.3 *μ*l cell^−1^ day^−1^ (varying by a factor of about four with the specificities of the CTLs). This value must be multiplied by a volume factor to obtain the whole-body parameter useful here; the factor is roughly 1/(5.50 · 10^6^): 5 × 10^6^ for the five liters of peripheral blood and 50 because only 2% of T cells reside in PB (98% residing in tissues), while the data analyzed in [11] referred to cells in serum. The rate of activation (labelled *α* in Materials and Methods) of resting cells, by professional antigen-presenting cells, is distinguished in our model from the rate that CTLs become activated and kill (labelled *κ*), by T cell receptor engagement with HLA-peptide complexes on target cells. Due to the lack of studies distinguishing these rates, we used the same value for both.

**Figure 1:**
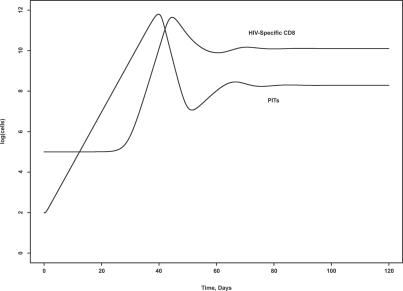
Whole-Body, Deterministic Model Suitable for HIV, Showing Infection and Immune Response; with *R*_0_ = 4, progression time (IT to PIT), one day; and *α = κ =* 10^−10^

With other choices of rate-constants, suitable for other pathogens targeting different tissues (e.g., influenza), the PP-model (programmed-proliferation model) can predict eradication after primary viremia [24,25] (see Figure 2). Key elements are a faster-growing pathogen (e.g., with a shorter eclipse period or larger *R*_0_) and higher immune system activation or killing rates. Hence, this conclusion is implausible for HIV (or SIV), at least based on currently available models.

**Figure 2:**
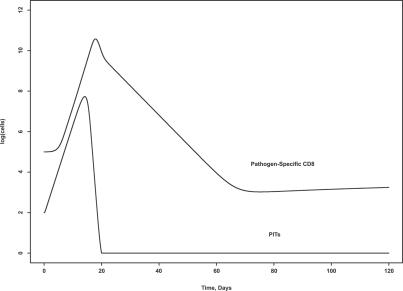
Whole-Body, Deterministic Model Suitable for a Faster, More Immunogenic Pathogen (Bacteria or Virus, but not HIV), with *R*_0_ = 5 Progession time, 1/4 day; *α* = 10^−5^; *κ* = 10^−9^.

#### Can T cells abort an HIV infection? The small-volume, stochastic model.

The prevention question involves different biology than that for eradication, as well as distinct modeling challenges. Concerning the biology, depending on the route of infection, the initial confrontation might be in blood, lymph nodes, or mucosal tissues. The concentration of memory T cells will differ in these compartments; as will the activation and killing rates. For the mucosal route, it is even conceivable that a special class of memory effector cells resides in mucosa which can kill immediately upon T cell receptor engagement but without requiring activation by antigen-loaded antigen-presenting cells. (The military analogy would be pickets, guarding against the enemy's advance units.) The basic reproductive number (*R*_0_) may be different from the value we adopted when discussing the global infection, because the concentration of target cells at the portal may differ from the whole [34]. For example, in rectal exposure to HIV, the gut is rich in activated, memory CD4+ T cells, the virus's primary target.

Several other observations about host and pathogen biology may be relevant to the prevention question. Virologists have noted that PITs produce a highly variable number of virions, in the range 50–1,000 per day in the productive period [35]; also, at least in vitro, only one virion in 10,000 is infectious (M. Emerman, personal correspondence to WDW). Rampant bottlenecking may characterize retroviral population dynamics; indeed, some HIV geneticists believe that the virus population in vivo has a low effective population size (“*N_e_*”) [36,37]. Geneticists can provide many explanations for a low *N_e_;* e.g., periodic selective sweeps, “bio-geographic” localization of lineages (i.e., in different tissues), or migration-extinction episodes. (Others doubt that it is low [38].) Yet another proposal, which we shall call “extra-Poisson variation” (EPV) and incorporate in our model, attributes the bottlenecking to the level of the individual PIT. Finally, HIV epidemiologists have ascertained that the risk of infection given exposure is surprisingly small, typically in the range 1/2,000 to 1/200 depending on the mode of transmission and the population studied [39–41].

Concerning modeling, two aspects of the prevention problem render the whole-body, deterministic model inapplicable. First, the site of the initial struggle may be confined. If so, the rate parameters for activation and killing must be suitably rescaled, because they reflect the probability of cells coming together. Consider, for example, one PIT, 10^9^ HIV-specific, CD8 T cells (1% percent of the CD8 compartment, an optimistic goal for a vaccine), and activation and killing rates of about 10^−10^ *μ*l cell^−1^ day^−1^. If we assume the cells are uniformly distributed in five liters of peripheral blood (or several kilograms of tissues), the whole-body, uniformly mixed model predicts that, as infectious virions are swept through blood and lymph, an activated CTL will soon appear—but the impact on the lifetime of the solitary PIT will be infinitesimal. The reason is that, due to the uniform mixing hypothesis, the CTL is likely to be far away (i.e., in another of the five million microliters of peripheral blood, or another lymph node). But the first CTL may appear, divide, and function nearby. Imagine a volume drawn around the initial PIT, just sufficient to enclose one HIV-specific CTL. Because vaccine designers have settled on 50 HIV-specific CTLs per 10^6^ peripheral blood mononuclear cells as an empirical criterion for an interesting immunogen, we imagine this volume in the range 2–20 *μ*l. The rate constants for activation and killing should be rescaled from the value estimated in [11] by the inverse of this volume—as opposed to the factor mentioned in the previous section. These rate-constants will be at least six orders of magnitude larger than suitable for the whole-body mixed model.

Starting from those two precursors, we can envisage a race between infection process and immune response. Modeling such a competition, with small numbers of players, by deterministic rate equations is out of the question. Both infection and immune dynamics must be considered stochastic and modeled by jump Markov processes. In similar situations, branching-type processes have appeared in modeling clonal extinction in multistep carcinogenesis [42] and the impact of drugs in primary HIV or SIV infection [23]. However, because of the interactions that appear in the activation and killing rate functions, our processes are not of the pure-branching type. For example, “daughters” of different “parent” CTLs are not independent, because they share in the activity of recognizing or killing the same PITs; likewise, the different PIT lineages are not independent because they stimulate and are killed by the same clone(s) of CTLs. No analytical or numerical techniques are available for computing the extinction or other probabilities (as for instance were exploited in [23]); we must rely on simulations. On the positive side, as we are only interested in outcomes with peak infection less than some small upper bound (e.g., 1,000 PITs), simulation is fast (and we do not need the sophisticated switchover techniques of [26,43].)

To allow for a dynamical explanation of a low effective population size, as well as the small infection probability given exposure, we amplified the basic infection model by assuming multiple types of PITs (as in [44]). A PIT of type “*k*” is generated with probability *p_k_* and produces virions at rate *v_k_*. Because absent this additional heterogeneity for a fixed PIT lifetime the number of its “offspring” is a Poisson random variable, we will refer to this class as exhibiting EPV. The simplest model (see Equation 11) has three PIT types, which produce none, one, or many (e.g., 1,000) offspring per unit time, with a two-parameter probability law arranged to yield the chosen degree of EPV and so that the third production level contributes to the mean (e.g., by setting, with *v*_3_ = 1,000 and mean = 3, *p*_3_ = 2.5/1,000). The connection with *N_e_* is explained in [44]. With these assumptions, the infection process may be dominated for a time by unlucky progenitors that have fewer than two offspring, which pushes the extinction probability close to one [44].

Hence, we chose the PIT-heterogeneity parameters to generate a low infection-given-exposure, although since extinction is a dynamic outcome the exact figure will depend on other parameters, especially the initial volume (*V*) and the basic reproductive number (*R*_0_). In Figure 3, we display the vaccine efficacy (VE) for preventing infection as a function of volume *V,* for several choices of *R*_0_. The initial condition was one PIT of type *k* with probability *p_k_* and, in vaccinees, one HIV-specific, resting memory cell. The stochastic process was halted if the infection compartment went extinct, exceeded 1,000, or the time reached 30 days; in the latter cases the infection was declared established. In a sizable fraction of cases, the infection attained more than 100 cells but was eventually eliminated, demonstrating that CTLs can win the race.

**Figure 3:**
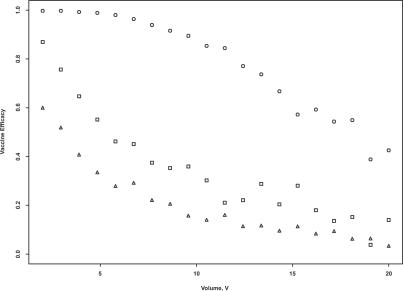
Vaccine Efficacy for Prevention as a Function of *V,* as Simulated from the Small-Volume, Stochastic Model for Three Values of *R*_0_ The EPV was moderate (see Table 1 footnote), yielding a probability of infection-given exposure that ranged from 0.01–0.2, depending on *V* and *R*_0_. 10,000 stochastic simulations were used to produce each point in this figure. Circles, *R*_0_ = 4; squares, *R*_0_ = 10; and diamonds, *R*_0_ = 20.

## Results

We simulated 500 trials under various hypotheses about vaccine action. A trial consisted of enrolling subjects and simulating the effect of one exposure per subject, until 100 infections were recorded. To generate a variety of outcomes reflecting the host and viral heterogeneity we would expect in any subject population, we randomized the resting-cell activation parameter (*α* in Materials and Methods in the section Modeling the Cellular Immune Response) over subjects, multiplying it by a log-normal random variable with mean one and standard error 2.0. As a consequence of the programmed-proliferation assumption (rendering the immune system a “high-Q” amplifier, in engineering language), the effect on the steady-state immune response is substantial and the viral load becomes distributed also as log-normal with deviation of several logs (see Figures 4 and 5), as has been observed in cohorts of patients in the chronic stage of infection [14]. For each subject, an initial stochastic simulation determined the infection status (at 30 days); in the infected case, it was followed by a deterministic simulation to compute the viral load (at 120 days). The initial conditions for the stochastic simulation were as described above, assuming a volume *V* = 15 *μ*l. We fixed the initial condition for the deterministic simulation at 100 ITs; as the model exhibits a stable steady state, this choice mattered little.

**Figure 4:**
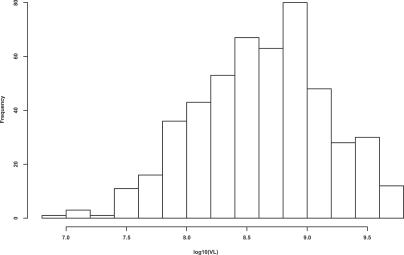
Histogram of Viral Loads, Generated from Variable Activation Parameter (*α*)

**Figure 5:**
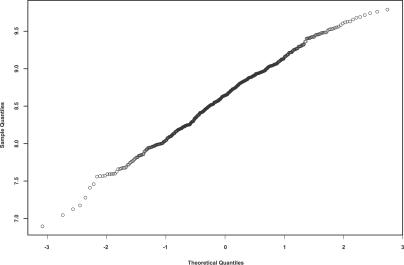
QQplot of Viral Loads, Generated from Variable Activation Parameter (*α*) against a Normal Law

Here we report on the performance of statistical estimation for three hypothetical vaccine scenarios. The initial conditions on the immune system refer to the deterministic simulation used to compute the viral load.

### Case 1.

The death rate of natural HIV-specific memory cells was .33 per day (“defective memory” scenario); there were 10^5^ naïve, HIV-specific CD8s, but no memory cells in either vaccinated or placebo subjects.

### Case 2.

The death rate of natural HIV-specific memory cells was .33 (“defective memory” scenario); there were 10^5^ naïve HIV-specific CD8s in both groups and 10^9^ vaccine-raised memory cells in vaccinated subjects but none in placebo subjects.

### Case 3.

The death rate of vaccine-induced HIV-specific memory cells was .00017 (“defective memory” cured); there were 10^9^ memory (but no natural) HIV-specific CD8 cells in the vaccinated subjects and 10^5^ naïve but no memory cells in unvaccinated subjects.

The true VE for prevention in each case was 0.51 (by simulation, using the stochastic model). In Case 1, there was no causal vaccine effect on viral load, and so any estimated effect must be due to selection bias. In Case 2, since the “defect” was not cured we expect to see only a modest effect on viral load. (Due to the stability of the steady state, effects of initial conditions are transient.) In Case 3, there was a large causal vaccine effect on viral load.

For each case, for each of the 500 vaccine trials, VE was estimated as one minus the ratio of proportions of vaccine and placebo recipients infected, and the *ACE*(*β*) was estimated using the GBH method for *β* = −1,0,1,2. For all three scenarios, the average VE estimate over the 500 trials was 0.57, close to the true 0.51. Figure 6 summarizes the results on estimation of *ACE*(*β*). For Case 1, the analysis that assumes no selection bias (*β* = 0) shows that the setpoint viral load is about a third log higher on average in infected vaccinees than in infected placebos. However, once possible selection bias is accounted for, the causal vaccine effect on viral load is seen to be consistent with nullity (*β* = 0 panel). Therefore, the GBH method leads to the conclusion of no causal vaccine effect, thereby protecting against spuriously concluding harm by vaccine. The methods applied to Case 2 provide a similar conclusion. In contrast, for Case 3 the estimated *ACE*(*β*) ranges from about 0.8 (at *β* = −1) to 1.5 (at *β* = 2), with the lower 95% confidence limits always exceeding 0.0, so that the method correctly concludes a beneficial causal vaccine effect to lower viral load, robust to plausible levels of selection bias. The GBH methods for estimating the causal effect of vaccine on viral load therefore pass these three tests posed by mechanistic, model-based, and simulated trials.

**Figure 6:**
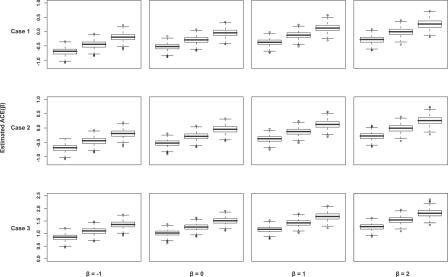
Boxplots of the *ACE*(*β*) Estimates For the 500 simulated STEP vaccine trials per case, each panel shows a boxplot of the *ACE*(*β*) estimates (middle boxplot), and boxplots of the lower and upper 95% confidence limits for *ACE*(*β*). (A–D) Case 1. (E–H) Case 2. (I–L) Case 3.

## Discussion

Novel causal inference methods will be applied to analyze the viral load primary endpoint in the first two efficacy trials of a T cell HIV vaccine. But the operating characteristics of these methods in plausible biological scenarios have not been studied. This article described novel mechanistic models of CTL and HIV kinetics which provided biologically grounded simulations of the STEP trials. We found that the causal methods provided inferences that accurately reflected the assumed mechanisms. The mechanistic models, in particular the small-volume, stochastic case, also yielded the (surprising) conclusion that memory T cells might, in a natural setting (as opposed to animal trials with huge inoculums), abort an HIV infection.

The process used to elicit a range of plausible values for the sensitivity parameter for estimating the *ACE* was as follows: Shepherd, Gilbert, and Mehrotra [unpublished data] sent a letter to 10 HIV vaccine experts, with approximately five pages of discussion of the interpretation of the sensitivity parameter, and asked each expert to provide their opinion on minimum and maximum plausible values. Eight of the ten experts responded. This information will be used in the analysis of the Merck/HVTN vaccine efficacy trial.

What are the advantages or disadvantages of GBH's method (via expert-elicited parameter) relative to other approaches to assessing efficacy of a T cell vaccine? A leading alternative primary analysis is to compare the “burden-of-illness” between vaccine and placebo recipients, including all randomized subjects in the analysis, and to assign a value of zero for the viral loads of uninfected subjects [45]. The advantage of this analysis is that it provides unbiased inferences on a causal effect. One disadvantage is that it aggregates the vaccine effects on infection and viral load, so that other methods that assess infection and viral load separately are needed to address mechanisms of protection. A second is that it has low power compared with the GBH causal inference approach that conditions on infection. The much lower power was documented in extensive simulations [13] and occurs because the majority of subjects are not infected during the trial. Both methods are, of course, free from questionable biological assumptions that are inevitable in any mechanistic model. Presumably no vaccine will be licensed based on model-based data analysis in the near future. On the other hand, models make explicit assumptions that can be criticised or accepted, unlike expert opinion whose basis may be unclear.

The many alternatives to our biological assumptions suggest new directions for research. The scenario we developed for prevention of infection in the Introduction linked a low effective population size to EPV in virion production and to the small observed probability of infection given exposure. An alternative explanation for the latter is stochastic breach of a mucosal barrier. In this scenario, presumably either a large number of or no PITs at the mucosal site would be expected at each exposure. The increased risk associated with genital ulcers or abrasions during sexual activity supports this theory, while the risk associated with non-ulcerative genital infections might be taken as supportive of the stochastic-extinction theory (by recruiting memory activated CD4s to the mucosal membrane, the STD would enhance the probability of making a “successful” PIT) [46].

Concerning that first PIT (or PITs), several groups have endeavored to determine the infection probability as a function of inoculum size in animal models [47–50]. With some additional model development and biological hypotheses, data from repeated low-dose exposures might be converted into information about the PIT distribution. In this regard, the observations of Ritola et al. [51], of several variants of the envelope protein making an appearance during primary infection are interesting. As the authors remark, the finding that two or three variants appear almost as frequently as one is inconsistent with a free-virion route and a low probability of infection (implying the Poisson law). However, Jung et al. [52] discovered that HIV-infected T cells harbor an average of 3.6 proviruses, frequently with distinct genomes; hence the observations of Ritola et al. [51] are consistent with infection by one initial cell. The assumption in the stochastic model of one initial PIT is of course the simplest; but if there are *N* initial PITs per exposure, distributed as a Poisson random variable with mean *I,* for example, and the outcomes of the different local infections are independent, the prevention probability becomes exp (I{*p*_ab._ − 1}), where *p*_ab_ is the (simulation-derived) probability of aborting an infection chain with one initial PIT. If, however, the infections are contiguous (e.g., at the same mucosal surface), the processes are not independent (because both activate and are recognized by the same CTLs), and the probability can only be learned through simulations.

With respect to model realism and simulation technique, when more data—and faster computers—become available, the compartmental design of the small-volume model should be replaced by an agent-based approach. One motivating factor is the unrealistic time-to-event distributions in Markov models—which must, by the assumed Markov property, be exponential. For modeling biology, this means that lifetimes have implausibly heavy tails, and the additional perverse effect that individuals die but cannot age. In our context, another peculiar consequence concerns the number of virions produced by a PIT, which we might expect to have a Poisson distribution—but, in Markov models, it must be geometric (because the PIT lifetime must be exponential). Thus, compartmental models already exhibit a (moderate) degree of EPV. Adding more compartments can partly restore realism, by converting exponentials to gammas [53]; but the heavy-tail problem persists. By contrast, in agent-based models, arbitrary time-to-event distributions can be incorporated; when interest centers on tail events (such as extinctions), changing over might have substantial impact. Indeed, we built an agent-based simulator for the small-volume stochastic model and noted—adopting a Weibull distribution, with exponent three (which has a much lighter tail) for all lifetimes—increased extinction rates. However, at present we lack data on the proper time-to-event distributions for T cell kinetics, and the agent-based routine is 100 times slower than for the compartmental model, rendering simulating thousands of trials a daunting task.

## Materials and Methods

### Estimating the *ACE* of vaccination on viral load.

We briefly present the statistical details of GBH's method for estimating the *ACE* of vaccination on viral load. The method is formulated using the potential outcomes framework for causal inference [32,54]. Let **Z** be the vector of vaccination assignments for the *N* randomized subjects, with *i*th element *Z_i_* (*Z_i_* = *v*, vaccine; *Z_i_* = *p*, placebo). Let **S(Z)** be the *N*-vector with *i*th element *S_i_*(*Z*), which is the indicator of whether the *i*th subject would be infected given **Z**. For subjects with *S_i_*(**Z**) = 1, let *Y_i_*(**Z**,**S**) be the potential viral load if assigned **Z**, given **S** = **S**(**Z**). We adopt Rubin's Stable Unit Treatment Value Assumption (SUTVA; [54]), which states that *S_i_*(**Z**) = *S_i_*(**Z**′) whenever *Z_i_* = 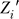 , and *Y_i_*(**Z**,**S**) = *Y_i_*(**Z**′,**S**′) whenever *Z_i_* = 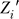 and *S_i_*(*Z_i_*) = 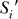 (*Z_i_*) = 1. SUTVA implies that the potential outcomes (*S_i_*(*v*),*S_i_*(*p*),*Y_i_*(*v*),*Y_i_*(*p*)) of each subject *i* are unrelated to the assignment *Z_j_* of other subjects, and allows *S_i_*(**Z**) and *Y_i_*(**Z**,**S**) to be written as *S_i_*(*Z_i_*) and *Y_i_*(*Z_i_*), respectively. Under SUTVA, each subject has two potential infection outcomes (*S_i_*(*v*), *S_i_*(*p*)) and at most two potential viral load outcomes (*Y_i_*(*v*),*Y_i_*(*p*)). For each subject, only one of *S_i_*(*v*) or *S_i_*(*p*) is observed, denoted 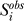 ≡ *S_i_*(*Z_i_*), and in the subgroup with 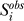 = 1, 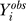 ≡ *Y_i_*(*Z_i_*) is observed. Note that *Y_i_*(*Z*) is defined only if *S_i_*(*Z*) = 1.

Any comparison between the ordered sets {*Y_i_*(*v*):*S_i_*(*v*) = *S_i_*(*p*) =1} and {*Y_i_*(*p*):*S_i_*(*v*) = *S_i_*(*p*) = 1} is a causal effect, because it is made within a subgroup of subjects with common joint values of (*S_i_*(*v*),*S_i_*(*p*)) (Property 2 in [32]). GBH referred to the subgroup {*S_i_*(*v*) = *S_i_*(*p*) = 1} as the “always-infected” principal stratum, as it comprises subjects who would be infected no matter whether they received vaccine or placebo. Let

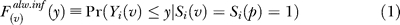


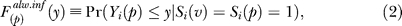
and assume the *Y_i_*(*v*) are identically distributed as 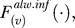 and the *Y_i_*(*p*) are identically distributed as 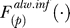 . Any functional that measures a contrast of 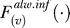 and 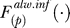 is a causal effect, and GBH focused on the *ACE* estimand, defined as


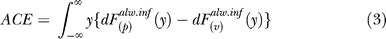


Because neither distribution in Equations 1 and 2 are identifiable (since *S_i_*(*v*) and *S_i_*(*p*) are not both observed), assumptions are needed to be able to estimate *ACE*. In addition to SUTVA, GBH made the following two assumptions for identifying the distributions:

**A1:** The assignment *Z_i_* of each subject is independent of his/her potential outcomes (*S_i_*(*v*),*S_i_*(*p*),*Y_i_*(*v*),*Y_i_*(*p*)).

**A2:** For each subject *i,* Pr(*S_i_*(*v*) = 1,*S_i_*(*p*) = 0) = 0.

Assumption A1 plausibly holds in HIV vaccine efficacy trials due to randomization and blinding. A2 states that no subject would be infected if randomized to vaccine but uninfected if randomized to placebo, and under A1 will hold if vaccination does not increase the per-exposure infection probability for any trial participant.

Assumption A2 implies that all infected vaccine recipients are in the always-infected principal stratum, so that 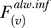 (*y*) = *F_v_*(*y*) ≡ Pr(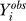 ≤ *y*|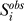 = 1,*Z_i_* = *v*), where *F_v_*(·) is the distribution of viral load in infected vaccine recipients; therefore 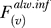 (·) is identified from the observed data. A1 and A2 do not identify 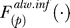 , however, because they do not determine whether an infected placebo recipient is in the “protected” {*S_i_*(*v*) = 0,*S_i_*(*p*) = 1} or always-infected {*S_i_*(*v*) = 1,*S_i_*(*p*) = 1} principal stratum. To identify 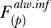 (·), GBH supposed a semiparametric model relating it to *F_p_*(·), the identified distribution of viral load in infected placebo recipients:

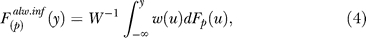
where *w*(*y*) = Pr(*S_i_*(*v*) = 1|*Y_i_*(*p*) = *y*,*S_i_*(*p*) = 1) and *W* = 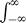 *w*(*u*)*dF_p_*(*u*) is a normalizing constant. The weight function w(*y*) is the probability that a subject infected with viral load *y* if randomized to placebo would be infected if randomized to vaccine. If *w*(·) were known, then both 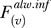 (·) and 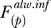 (·) would be identified, and the *ACE* could be estimated. However, *w*(·) is unknown, and the data plus A1–A2 do not inform about whether a particular *w*(·) is correctly specified. Accordingly, GBH assumed *w*(·) is a known cumulative distribution function, and recommended estimating *ACE* for a variety of fixed choices of *w*(·) as a form of sensitivity analysis. GBH focused on *w*(·) equal to the inverse logit function, *w*(*y*) = *w*(y | *α*,*β*) = exp{*α* + *βy*}/(1 + exp {*α* + *βy*}), which allows *w*(·) to be constant or smoothly monotone increasing or decreasing. For *β* finite, exp(*β*) is the odds ratio of infection under randomization to vaccine given infection under randomization to placebo with viral load *y* versus with viral load *y* − 1. This interpretation allows the choice of *β* to be guided by beliefs about plausible degrees of selection bias.

Given fixed *β, α* is determined as the solution to the equation 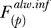 (∞|*β*) = 1. Fixing *β* = 0 specifies a constant weight *w*(*y* | *α*,*β* = 0) = *W,* and reflects an assumption of no selection bias. Fixing *β* > 0 makes w(y | *α*,*β*) monotone increasing in *y* and reflects “positive” selection bias, with infection odds under randomization to vaccine higher for a larger potential viral load *Y*(*p*) = *y*. Similarly, *β* < 0 makes *w*(*y* | *α*, *β*) monotone decreasing in *y* and reflects “negative” selection bias, with infection odds under randomization to vaccine lower for a larger *y*. Ranging *β* from −∞ to ∞ spans all possible magnitudes of selection bias parametrized by *w*(·).

To describe the estimator of *ACE*(*β*) developed by GBH, let *n_v_*(*n_p_*) be the number of infected subjects in the vaccine (placebo) arm, and *Y_v_*_1_, … , *Y_vn_v__*(*Y_p_*_1_) … *Y_pn_p__*) be the viral loads in infected vaccine (placebo) recipients. For a given *β,* the semiparametric maximum likelihood estimator 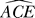 (*β*) of *ACE*(*β*) is given by

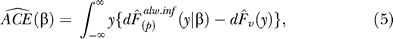
where 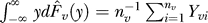 and 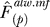 (*y* | *β*) is the maximum likelihood estimator of 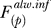 (*y* | *β*) under the model described in Equation 4, calculated as

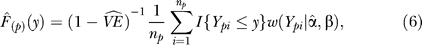
where 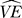 is the maximum of zero and one minus the ratio of infection rates (vaccine/placebo), and 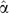 is computed as the solution to the equation 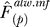 (∞ | *β*) = 1 as a function of *α*. Semiparametric bootsrap percentile confidence intervals about *ACE*(*β*) can be computed by resampling from 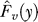 and 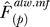 (*y* | *β*). Computing point and interval estimates of *ACE*(*β*) for a range of fixed values of *β* constitutes a sensitivity analysis. We used 500 bootstrap samples.

#### Modeling the infection process.

We used a simplified version of the compartmental model, proposed in [24], adapted, in the stochastic case, to incorporate various types of infected cells. (Only one type is required for the deterministic model.) Each type has two life stages. The first stage, labelled *X_k_,* where *k* denotes the type, represents the count of IT or “eclipse”-phase cells, before virions appear, and the second, labelled *Y_k_,* the count of PIT cells. The possible jumps and rates are described in Table 1.

In the presence of the immune response, *δ_PIT_* (inverse PIT lifetime) was modified to *δ_PIT_* + *κCTLs,* where *CTLs* and *κ* are defined as in the next section, Modeling the Cellular Immune Response. We derived the infection rate *ι* from the basic reproductive number, *R*_0_:


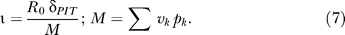


With this definition, the growth rate of infection (sum of *X*s and *Y*s) is governed by the eigenvalue (let *δ* = *δ_PIT_*):


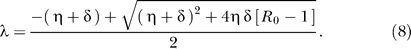


We chose *R*_0_ in the range 4–6 (except in Figure 3, which explores a wider span), which with the other parameters in Table 2 yields a doubling time (log2/*λ*) in the range 0.88–1.3 days, and time-to-peak viremia (assuming 7 logs growth) of 20–30 days, consistent with observations of HIV before the peak [56,57]. (One investigation, [56], reported a much shorter doubling-time at early timepoints, and much larger *R*_0_, but these estimates were derived from an extrapolation using a target cell–limited rather than an immune-control model.) As our goal is to investigate the statistical properties of methods that assume CTL control, we adopt the latter. For other discussions of TCL and IC in primary infection, see [9,58]. In the statistical study we used *R*_0_ = 4, as it creates the maximal bias and the largest opportunity for the method to fail.

For the production rates, with three types (the simplest non-trivial case), we usually chose *v*_1_ = 0, *v*_2_ = 1, *v*_3_ = *K,* and


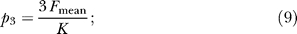



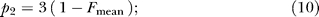



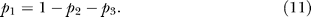


Here *F*_mean_ is the fraction of the mean contributed by the third type. Choosing *F*_mean_ to be nearly one avoids trivial cases where the third type contributes infinitesimally to the infection rate. Note that this construction yields mean virion production *M* = 3 independent of *K* and *F*_mean_. Table 2 records our parameter values for the infection process.

#### Modeling the cellular immune response.

Again, we used a simplified version of the compartmental model introduced in [24]. Let *W_i_* denote HIV-specific CD8 compartments. The allowed transitions are described in Table 3.

Let *NR* be the index of the naïve resting compartment; *MR,* the memory resting compartment; and *n_d_* the number of permitted doublings. In the jumps labelled mitoses, the index ranges over *i* = *NR*, …, *NR* + *n_d_* − 1 or *MR*, …, *MR* + n*_d_* − 1; in the jumps labelled deaths, *i* = *NR*,*MR*,*NR* + *n_d_* or *MR* + *n_d_*; in the jumps labelled reversions, *i* = *NR* + *n_d_* − 1 and *j* = *MR*. Activation into the cell cycle of resting, naïve, or memory CD8s occurs at rate *ν_NR_* = *α PITS* or *ν_MR_* = *α*[memory-factor] *PITs*, where “PITs” denotes ∑ *Y_k_* (from the previous section “Modeling the infection process”); otherwise, *ν_i_* = 1/ (cell-cycle time). The CTLs appearing in section 1 are the sum of W_i_ for *i* = *NR* + 4, … *NR* + *n_d_* (naïve CD8s promote to effector status after four divisions) or *i* = *MR* + 1, … *MR* + *n_d_* (memory promote after one). We set *ζ* (thymic immigration of resting, naïve HIV-specific CD8s) = *W_NR_* (at time zero) × *δ_NR_*, implying a steady-state immune system, absent activation. When only vaccine-induced cells are involved, as when simulating the infection probability, the naïve compartments (*W_i_,* for *i* = NR, … ,*NR* + *n_d_*) are deleted from the model. We summarize our immune system parameters, which assume an HIV infection, HIV-specific CD8s, and the “defective memory” scenario, in Table 4. See [24] for the scenario and references to the literature.

#### Simulating from the models.

When employing the deterministic models, we simulated from the ODEs defined by


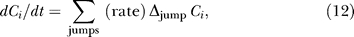


Here *C_i_* = *X_i_*, *Y_i_*, or *W_i_* is any model compartment and Δ is the difference operator corresponding to the jump. To update in the deterministic model, we used a time-step of .01 day and fourth-order Runge–Kutta. We simulated the jump processes (used for computing the infection probability) by the “direct” method [43]: drawing exponential waiting times for all transitions according to the (inverse) rates at each time; determining which was the next jump; implementing it; then updating all rates. The program was written in C language and is available from the first author. Simulating 500 trials required about three days using one processor (a 3.06-GHz, Intel Xeon) of a Pentium workstation.

## **Abbreviations:**

*ACE*: average causal effect
CTLs: cytotoxic T lymphocytes
EPV: extra-Poisson variation
GBH: Gilbert, Bosch, and Hudgens
IT: infected target
PIT: productively infected target
SIV: simian immunodeficiency virus
SUTVA: Stable Unit Treatment Value Assumption
